# Evaluation of the Accuracy of Liquid-Based Oral Brush Cytology in Screening for Oral Squamous Cell Carcinoma

**DOI:** 10.3390/cancers11111813

**Published:** 2019-11-18

**Authors:** Lena Deuerling, Kristin Gaida, Heinrich Neumann, Torsten W. Remmerbach

**Affiliations:** 1 Section of Clinical and Experimental Oral Medicine, University of Leipzig, Liebigstraße 10-14, 04103 Leipzig, Germany; Lena.Deuerling@t-online.de (L.D.); birthe-kristin@t-online.de (K.G.); 2 Medical Care Center for Histology, Cytology and Molecular Diagnostics, 52351 Düren, Germany; heinrich.h.neumann@gmail.com; 3 German Association of Oral Diagnostics (DGOD mbH), Wettiner Str. 10, 04105 Leipzig, Germany

**Keywords:** oral cancer, squamous cell carcinoma, brush biopsy, liquid-based cytology

## Abstract

This study evaluates the accuracy of the results of liquid-based oral brush cytology and compares it to the histology and/or the clinical follow-ups of the respective patients. A total of 1352 exfoliated specimens were collected with an Orcellex brush from an identical number of oral lesions, then cytological diagnoses were made using liquid-based cytology. The final diagnoses in the study were 105 histologically proven squamous cell carcinomas (SCCs), 744 potentially malignant lesions and 503 cases of traumatic, inflammatory or benign hyperplastic oral lesions. The sensitivity and specificity of the liquid-based brush biopsy were 95.6% (95% CI 94.5–96.7%) and 84.9% (95% CI 83.0–86.8%), respectively. This led to the conclusion that brush biopsy is potentially a highly sensitive and reliable method to make cytological diagnoses of oral neoplasia. The main advantage of a brush biopsy over a scalpel biopsy is that it is less invasive and is more tolerated by the patients. Therefore, more lesions can be screened and more cancers can be detected at an early stage.

## 1. Introduction

Squamous cell carcinomas (SCCs) are one of the most ubiquitous cancers in the world. In 2014, SCCs of the oral cavity and pharynx were among the 15 most common cancers in Germany [1]. Although a lot of research has been done, the full process of the development of SCCs remains unclear. Multiple abnormal molecular genetic events in various chromosomes and genes that are involved in the regulation of the cell cycle were found [2,3,4]. They can lead to dysfunction in the cell growth cycle and the mechanisms to repair or eliminate cell damage [5].

It has been well known for years that there are certain factors that correlate with oral SCCs, such as tobacco smoking, alcohol drinking and betel quid chewing [6,7,8,9,10,11]. There is some evidence that suggests there is a correlation between the human papilloma virus (HPV) and SCCs, especially for HPV 16 and 18, although this link has not yet been proven and is still undergoing research [12,13]. There are also a few oral lesions that have the potential to become malignant and where SCCs are more likely to develop [14,15]. These potentially malignant oral lesions include leukoplakia, erythroplakia and palatinal lesions in reverse smokers; furthermore, there are diseases that count as precancerous conditions, such as lichen planus, submucous fibrosis, actinic keratosis and discoid lupus erythematosus [14,16,17]. 

Potentially malignant oral disorders need to be diagnosed and treated early to prevent the progression to SCC. Although there have been improvements in the treatment of SCCs of the oral cavity, the survival rate of this tumour entity remains poor. The five-year survival rate is about 57%, meaning that nearly half of the patients die within five years of diagnosis [18]. However, if the detection and treatment of oral SCC happen at an early stage, mortality and morbidity rates drop and a full recovery is more likely [19,20]. Therefore, it is important to develop methods as accurate as possible in order to detect early-occurring abnormalities in cells.

One such method is exfoliative cytology, which is a minimally invasive method to collect cells from the oral mucosa [21,22,23,24]. Inaugurated in 1999, brush biopsy has only recently become the focus of scientific research [25,26]. The brush is easy to use, efficient in cell collection, relatively painless and well-accepted by patients [27,28]. 

Since a huge variety of oral lesions are known these days, it is impossible, in most cases, to obtain a final diagnosis with only a clinical examination. Further investigation is needed to avoid misdiagnosis and therapeutic errors [29]. Some dentists in Germany use brush biopsies as a first method for examining suspicious oral lesions to find out whether further examination will be needed. Most dentists do not perform a scalpel biopsy themselves and therefore, brush biopsy can be used to decide on the further process and avoid unnecessary invasive scalpel biopsies. Since 2004, compulsory health insurance in Germany monetarily compensates the use of brush biopsies in dental practices.

The importance of brush biopsy as a method of oral exfoliative cytology has been and still is discussed in various studies. Most of them seem to analyse the same questions: is brush biopsy sufficient to be used as a standard method for dealing with suspicious oral lesions? Is the statement about malignancy or non-malignancy reliable enough? Is it good enough to even replace other methods that have been considered the “gold standard” for years, such as scalpel biopsy and histology [30]?

In a previous study, we showed that brush cytology combined with the conventional smear technique has a high sensitivity (91.3%) and specificity (95.1%) for oral SCC [31]. Later we reported on a split-sample study comparing the results of conventional smears and liquid-based cytology (LBC) [32]. No significant differences in sensitivity and specificity were found, but LBC turned out to be the easier, quicker and more standardized procedure. Consequently, since June 2012, we have used liquid-based cytology for all the samples taken in our institution.

In this article, we retrospectively analysed the results of liquid-based brush biopsies of patients who were treated in the Section of Clinical and Experimental Oral Medicine, University of Leipzig, between June 2012 and February 2018. The differences between conventional cytology and liquid-based cytology were then discussed.

## 2. Results

From June 2012 to February 2018, a total of 1359 samples were collected. Seven samples (0.05%) were insufficient for cytological diagnosis and therefore were excluded from further analysis. Consequently, a total of 1352 samples from 992 patients were included in this study. A total of 55% of the study population were women (744 cases) with a mean age of 63.5 years, and 45% were men (608 cases), with a mean age of 59.1 years (Table 1). The mean age of all patients was 61.6 years. The final diagnoses in the study were 105 histologically proven SCCs, 297 cases of leukoplakia, 20 cases of proliferative verrucous leukoplakia, 29 cases of erythroplakia, 260 cases of lichen planus, 138 cases of lichen erosivus and 503 cases of traumatic, inflammatory or benign hyperplastic oral lesions (Table 2). These diagnoses occurred mainly on the alveolar ridge (30.3%), the buccal mucosa (28.4%) and the lateral border of the tongue (22.4%) (Table 3). From 105 diagnosed SCCs, 66% (69 cases) were seen in men, mostly older than 40, with a peak around 50 years of age. The rest of the diagnosed SCCs were in women (36 cases) over 40, with two peaks at 65 and 75 years. Most SCCs were seen on the border of the tongue (32.4%) and on the alveolar ridge (28.6%) (Table 3). Interestingly, it was found that more men than women had SCCs, and on average, they occurred at a younger age in men. 

In this study, the sensitivity for the detection of cancer cells using brush biopsy was 95.6% and the specificity for the detection of non-neoplastic cells was 84.9% (Table 4). The recorded positive predictive value was 36.6% and the negative predictive value was 99.5%. The brush biopsy results gave a ‘positive’ diagnosis in 89 cases and of these, 77 cases were histologically proven carcinomas and 12 cases turned out to be a false positive. Around 70% of the false-positive results were potentially malignant lesions. The brush gave a diagnosis of ‘suspicious’ in 56 cases, 15 of which turned out to be a carcinoma after further investigation. In total, 150 cases had a diagnosis of ‘doubtful’ and 16 of those cases were found to be a carcinoma. There were 1057 cases with a diagnosis of ‘negative’ and 1052 of them had no carcinoma. In five out of the 1057 cases, after performing a pathological investigation, a carcinoma was found in the follow-up period three weeks after the cytological diagnosis (Table 5). Nine dysplasias were found: three mild, one moderate and five severe. One mild dysplasia and two severe dysplasias had a ‘doubtful’ brush biopsy result, one severe dysplasia had a ‘suspicious’ result, and two mild dysplasias, one moderate dysplasia and two severe dysplasias had a ‘positive’ result. 

## 3. Discussion

In general, brush biopsy is non-invasive, relatively painless and is well-accepted by patients [28,33]. Compared to a scalpel biopsy, it is quicker and easier to perform. Patients prefer brush biopsy to scalpel biopsy because no local anaesthetic, scalpel instrumentation or suturing is needed [27]. In this study, the brush biopsy was combined with liquid-based cytology (LBC). After collecting cells from the oral mucosa, the brush was put into a preservative liquid and transported to the laboratory where slides were prepared. This procedure is different to the conventional preparation of brush biopsies which was used before the liquid-based preparation and is often still used. In the conventional preparation, multiple exfoliated cells are spread out on multiple glass slides immediately after the collection of the cells. The advantages of LBC were that there were fewer airdried artefacts and less contamination with imprecise elements such as blood or debris [21,34,35]. Furthermore, using theliquid-based preparation, it is possible to perform additional analyses such as DNA image cytometry with the same sample of cells [27,36].

Navone et al. (2007) also claimed that LBC has better results in sensitivity and specificity than conventional preparation. In total, 473 patients with SCCs or potentially malignant lesions were examined at the Oral Medicine Section of the University of Turin [36]. Eighty-nine samples were processed with conventional cytology and 384 with liquid-based cytology. They found the sensitivity and specificity for conventional preparation to be 85.7% and 95.9% and for LBC 95.1% and 99% [36]. Dolens et al. reviewed 14 articles to evaluate the effectiveness of cytopathology in diagnosing oral lesions [37]. Using conventional exfoliative cytology, they had a sensitivity and a specificity of 92% and 97%, repectively. For the liquid-based method, they showed a slight increase in sensitivity and specificity of 97% and 98%, repectively. Both studies showed that LBC has a higher sensitivity and specificity than the conventional method. Macey et al. (2015) reviewed diagnostic tests for oral cancer and potentially malignant disorders (PMD), including the results of twelve studies using brush cytology, and found both an average sensitivity and specificity of 91% [26]. Looking at the twelve studies included in the review, only four studies found a higher sensitivity than this present study, whereas ten studies found a higher specificity [26]. Remmerbach (2006) compared the results of 1074 conventional brush biopsies with the histologically proven diagnoses [30]. That study showed a sensitivity of 97.17% and a specificity of 90.84% for brush biopsies using the conventional method of preparation. In total, six out of 1074 cases showed a false-negative result; that is, the outcome of the brush biopsy was negative for tumour cells, but they turned out to be a carcinoma. Remmerbach et al. (2017) compared liquid-based and conventional cytology in a split-sample study with 113 cases [32]. The liquid-based method reached a sensitivity of 97.5% and therefore, was slightly better than the conventional method with a 96.3% sensitivity. Burkhardt and Schwarz-Furlan (2018) introduced the cell block method as another method to process cells from brush biopsies by using a “pocket of gelantine” [38]. They achieved a significant value of diagnostic accuracy; further research will be needed for the validation of the process.

This present study using brush biopsies with liquid-based cytology included 1352 samples. The sensitivity for the detection of cancer cells was 95.6% and the specificity was 84.9%. Comparing the results of this study with Remmerbach (2006), each representing a different form of preparation, shows that there is only a small difference in sensitivity and specificity between conventional and liquid-based preparation. However, the amount of brush biopsies performed per lesion was greatly reduced. Using the conventional method, Remmerbach (2006) performed five brush biopsies which were distributed over five glass slides. In contrast, this present study, using the liquid-based preparation, performed only one brush biopsy per lesion. Despite the slight decrease in sensitivity from the conventional method compared to LBC of around 1.6 percentage points, the whole process is simpler and requires less time. 

There were five cases out of 1352 with a ‘false-negative’ result in this present study. One case was found to be a different carcinoma rather than a SCC, which the brush biopsy did not test for in this study. Another was shown to be positive with an alternative diagnostic method. Although there was a slight decrease in false negative results when using liquid-based instead of conventional preparation following Remmerbach (2006), there still remained a significant number of false-negative results. The fact that false-negative results can lead to undiagnosed carcinomas worsening, possibly fatally, due to no further treatments being undertaken, means that the brush biopsy needs further improvements before it can become a fully reliable method on its own. Since the diagnoses of ‘suspicious’ and ‘doubtful’ do not lead to a clear result like ‘positive’ and ‘negative’, there is necessarily a decrease in the sensitivity and specificity. 

In this study, the data were initially analysed with a split between positive and negative. To that end, any ‘suspicious’ result was considered as ‘positive’ in terms of further action required, and likewise ‘doubtful’ was considered ‘negative’. It turned out that this bifurcation was rather ineffective in minimising the number of false negatives. To fix this, any non-negative result was then considered to be positive. That is, ‘doubtful’, ‘suspicious’ and ‘positive’ were all considered to be positive with regards to the final diagnosis. Clinically, both ‘suspicious’ and ‘doubtful’ should be understood as a warning to the potential existence of malignant cells where further investigation is required. This interpretation of oral specimens follows the German guidelines from Freudenberg and Böcking (1998), which were developed for the assessment of specimens from the extragenital region [39]. Other research groups suggested the adoption of a modified Bethesda Cytology system to oral specimens to define an international standardization [22]. Both approaches, as well as our assumption that all non-negative results should be interpreted as ‘positive’, require independent trials with identical criteria to verify the scoring. 

Kujan et al. (2018) called liquid-based cytology in combination with the Orcellex brush, which was used in this study, an “appropriate choice” with “considerable potential for early detection of oral cancer and potentially malignant disorders” [33]. 

## 4. Materials and Methods

**Figure 1:**
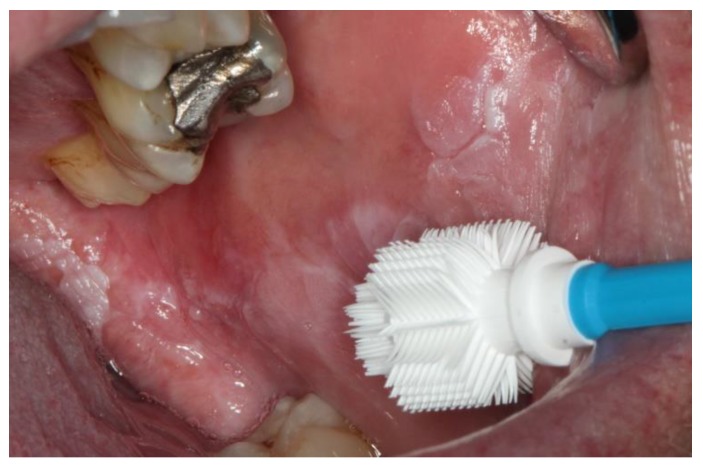
Cell collector Orcellex brush in front of a leukoplakia on the buccal mucosa.

**Figure 2:**
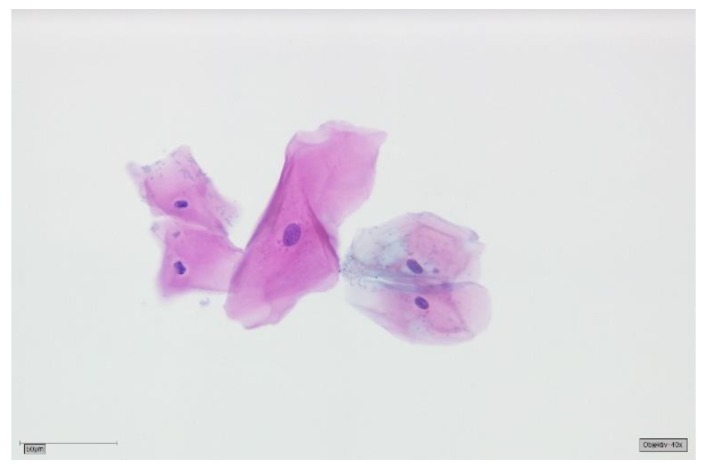
Negative for tumour cells—SurePath, staining Papanicolaou, lens 40×. Clinically most probably oral lichen planus (OLP). The background is completely clear. Bacterial flora can still be appreciated clinging to the cell surfaces. A group of five mature squamous cells with reactive changes: cytoplasmic hypereosinophilia and amphophilia, and megalocytosis of the central cell with corresponding mild nuclear enlargement. Small perinuclear halos of the two cells in the left field. All nuclei are round to ovoid with smooth contours and finely granular, evenly dispersed chromatin.

**Figure 3:**
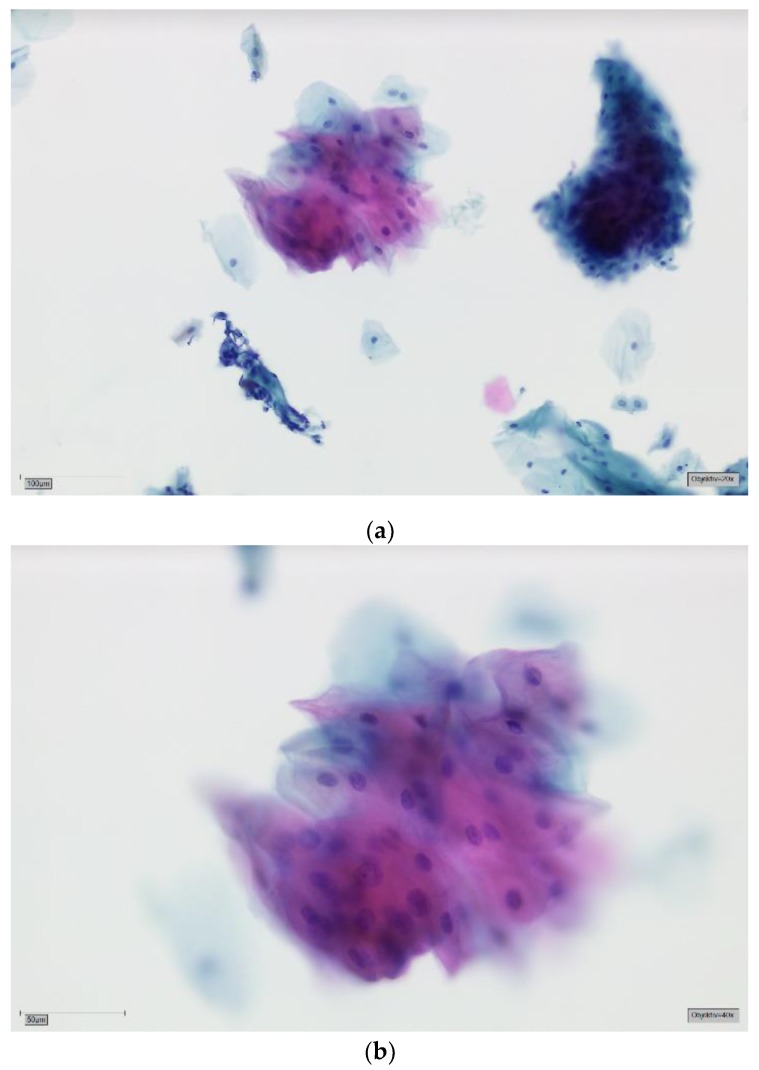
(**a**) Doubtful for tumour cells—SurePath, staining Papanicolaou, lens 20×. Clinically erosive OLP. The background is mostly clear. In the lower left field is a stromal tissue fragment with mechanically altered nuclei; this correlates with an erosive process. There are several mature squamous cells lying singly and three larger groups of cells. In the upper right field, the basophilic cells are more immature, a sign of regeneration. The group of mature cells in the upper central field shows cytoplasmic hypereosinophilia and amphophilia. There seems to be some degree of anisonucleosis. (**b**) Doubtful for tumour cells—detail, lens 40×. On high power and in this plane of focus marked variation in nuclear size can be appreciated (factor 2–3). The larger nuclei are rather darker, i.e., more hyperchromatic, and the chromatin is slightly coarse. Nuclear contours are still fairly smooth. These findings are commonly reactive. A mild squamous intraepithelial neoplasia (SIN1) cannot be excluded. We perform DNA-karyometry on these cases. DNA-aneuploidy should prompt invasive biopsy for histology. With DNA-euploidy we would recommend clinical follow-up and repeat brush cytology after 12 months.

**Figure 4:**
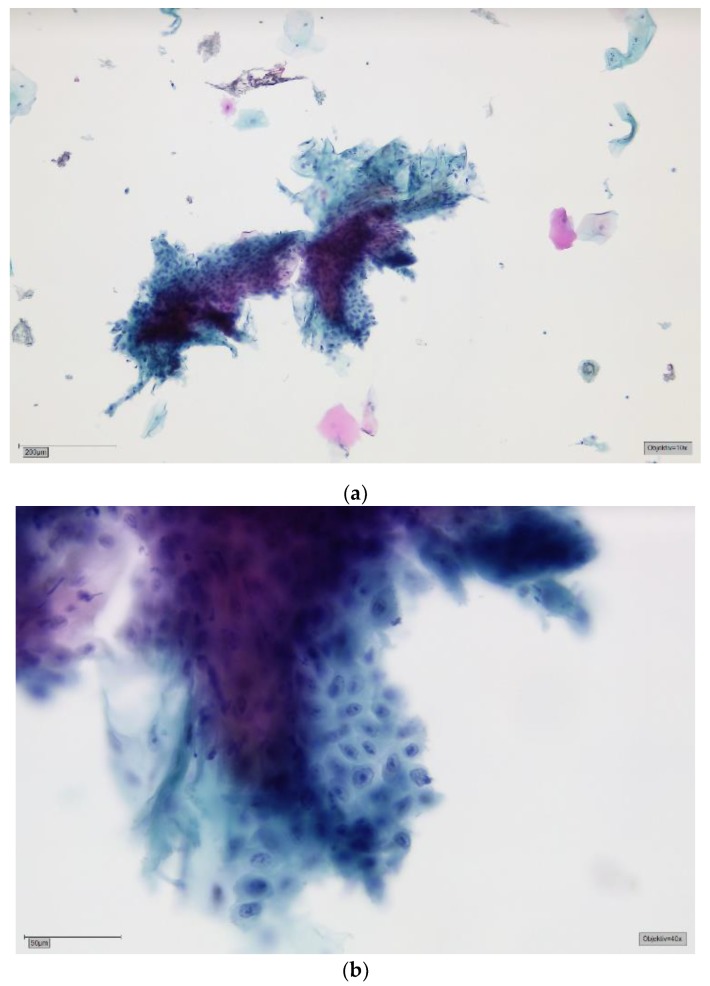
(**a**) Suspicious for tumour cells—SurePath, staining Papanicolaou, lens 10×. Clinically, an ulcerative lesion of the lateral border of the tongue. The background shows some amorphous deposits of partly eosinophilic, partly basophilic material, most obvious in the top central field. The big cell groups form partly sheets, partly three-dimensional crowds. Immaturity, a high nuclear/cytoplasmic (N/C) ratio and anisonucleosis can be suspected in this magnification. (**b**) Suspicious for tumour cells—detail, lens 40×. On high power there is marked anisonucleosis. The nuclei are haphazardly orientated, the axes of different nuclei are not parallel. Many nuclei show prominent nucleoli and/or irregularities of their borders. Chromatin is frequently irregularly deposited with early condensation along the nuclear membrane. These changes may represent so-called atypical tissue repair. The differential is high grade SIL or invasive SCC. The background may represent the ulcer or tumour diathesis. We would try to confirm the suspicion with DNA-karyometry. A scalpel biopsy must be performed for confirmation.

**Figure 5:**
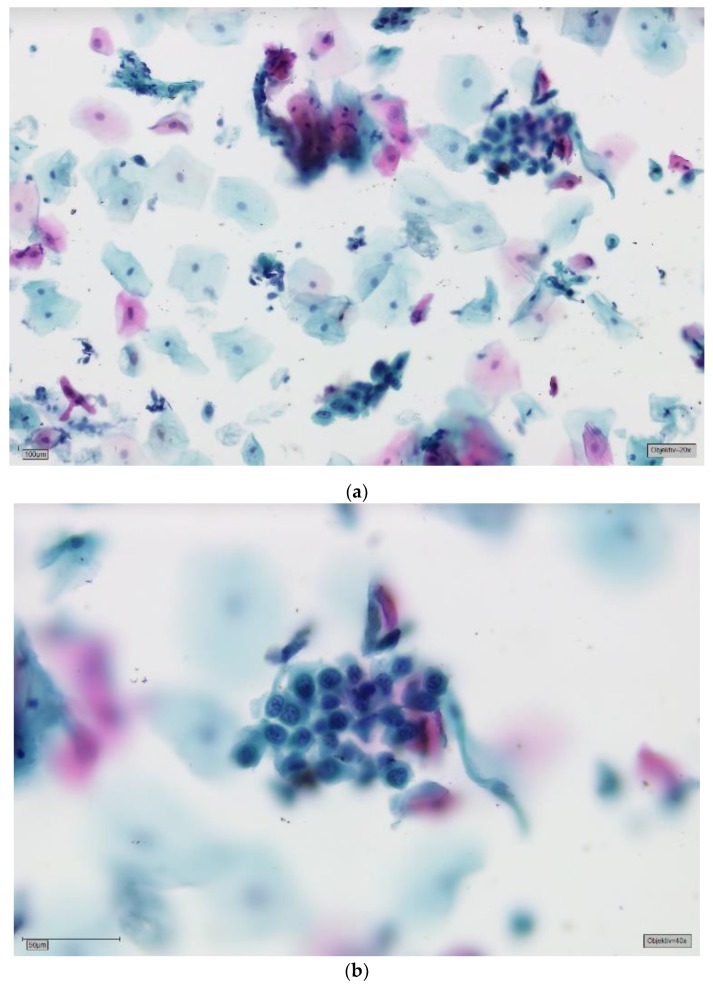
(**a**) Positive for tumour cells—SurePath, staining Papanicolaou, lens 20×. Clinically, there is suspicion for a carcinoma. On low power, there is a highly irregular aspect of the slide. In the background there are deposits of amorphous material, sometimes with nuclear fragments. In addition to many mature cells with normal nuclei, there are immature, small epithelial cells, both singly and in small and large groups. Nuclear enlargement and prominent nucleoli can be seen in this magnification. Some bizarre orangeophilic cells represent atypical keratinization. Those cells may have opaque, nearly black nuclei with smudged chromatin. (**b**) Positive for tumour cells—detail, lens 40×. A loosely cohesive sheet of highly atypical, immature squamous cells. The N/C ratio is markedly increased. Nuclei are highly hyperchromatic and chromatin is coarse. Some cells show an irregular nuclear contour and/or small nucleoli. These findings constitute the cytological diagnosis of a moderately well differentiated, keratinizing squamous cell carcinoma (SCC). Almost all these cases show aneuploidy on DNA-karyometry.

The patients had been referred to the Section of Clinical and Experimental Oral Medicine for diagnosis, treatment and examination of oral lesions. Samples were collected from the oral cavity by experienced members using the Orcellex brush (Rovers Medical Devices B.V. Oss, The Netherlands; Figure 1) between June 2012 and February 2018, together with liquid-based preparation. After twisting the brush ten times, the head of the cell collector was transferred directly into an alcohol-based liquid in the BD SurePath™ Collection Vial (BD Diagnostics—TriPath, Erembodegem, Belgium) and transported to the laboratory. In the Institute of Pathology (Medical Care Centre for Histology, Cytology and Molecular Diagnostics, Düren, Germany), the preparation of the preserved samples followed the instructions for SurePath™ preparations. Cells were transferred into a settling chamber on the slide where gravitational force lead to the different sedimentations of the cells. The slide was then stained according to Papanicolaou. After cleaning the slide with Xylene (or a Xylol-like fluid), the cells on the thin layer that were within a circle of a diameter of 13 millimetres were examined by an experienced cytopathologist and classified as either negative (Figure 2), doubtful (Figure 3a,b), suspicious (Figure 4a,b) or positive for tumour cells (Figure 5a,b), or insufficient for a result [39]. A ‘positive’ diagnosis means that malignant cells were found in the smear, ‘suspicious’ that abnormal cells with a vague sign of malignancy were seen, ‘doubtful’ that abnormal cell changes potentially occurred and for ‘negative’ no tumour cells were found in the smear [39]. In this study, non-negative results were considered to be ‘positive’, meaning that ‘suspicious’ and ‘doubtful’ were also treated as ‘positive’. This study was in accordance with the ethical standards of the ethics committee of the University of Leipzig (Ref.-No. 1272002) and with the 1964 Helsinki Declaration and its later amendments or comparable ethical standards.

This study consisted of 1352 cytological diagnoses performed on 992 patients, 440 of whom were men (44%) and 552 women (56%). Seven additional smears showed an insufficient number of cells, for which it was impossible to perform a cytological diagnosis and thus were not included. A total of 187 patients had multiple smears done over the years as their lesions were monitored in a clinical follow-up. The establishment of diagnostic accuracy was a clinical follow-up and pathohistological examination of a biopsy in some patients. In some cases, no further follow-up was performed because the clinical examination showed a minor temporary lesion like Aphthous stomatitis.

## 5. Conclusions

As shown in this study, brush biopsy is a non-invasive and well-tolerated method for screening suspicious lesions in the oral cavity. Despite similar sensitivities and specificities, liquid-based cytology is an improvement on the conventional method because the entire process is easier and takes less time. Even though the diagnostic accuracy of brush biopsy itself is high, further improvement could be achieved by combining it with DNA image cytometry as an additional method of investigation. Although it does not fully replace the gold standard scalpel biopsy and histopathological examination, brush biopsy has an important role in examining early oral lesions and screening potentially malignant lesions. The advantages of brush biopsy are that it is very easy to handle for the performing dentist, so more lesions may be screened, and more cancers might be found at a curable stage, meaning that there is a wider net for early detection.

## Figures and Tables

**Table 1:** Total numbers of sample size and average age (years).

Result Brush	Sample Size and Average Age (Years)		
	Male	Female	Total
Positive	63	26	89
	59.3	67.7	61.7
Suspicious	23	33	56
	67.9	66.9	67.3
Doubtful	65	85	150
	60.3	69.0	65.3
Negative	457	600	1057
	58.5	62.4	60.7
**Total**	608	744	1352
	59.1	63.5	61.6

**Table 2:** List of diagnoses and their frequencies.

Diagnosis	Frequency	
	Absolute	Relative
SCC	105	7.8%
Lichen	260	19.2%
Lichen erosivus	138	10.2%
Leukoplakia	297	22.0%
Proliferative verrucous leukoplakia	20	1.5%
Erythroplakia	29	2.1%
Other Lesions	503	37.2%
**Total**	1352	

**Table 3:** Location of all lesions and squamous cell carcinomas (SCCs) and their frequencies.

Location	Frequency All Lesions		Frequency SCC	
	Absolute	Relative	Absolute	Relative
Border of tongue	303	22.4%	34	32.4%
Back of tongue	26	1.9%	0	0%
Floor of the mouth	79	5.8%	16	15.2%
Buccal mucosa	384	28.4%	8	7.6%
Tonsil	8	0.6%	2	1.9%
Lip/Labial mucosa	37	2.7%	4	3.8%
Palate	106	7.8%	11	10.5%
Alveolar ridge	409	30.3%	30	28.6%
**Total**	1352		105	

**Table 4:** Diagnostic accuracy of brush biopsy.

Test	Accuracy	Confidence Interval (CI)	Lower Endpoint	Upper Endpoint
Sensitivity	95.6%	95%	0.945	0.967
Specificity	84.9%	95%	0.830	0.868
Positive predictive value	36.6%	95%	0.340	0.392
Negative predictive value	99.5%	95%	0.992	0.999

**Table 5:** Results of brush biopsy versus final diagnosis.

Brush Result	Cancer Diagnosis		
	Positive	Negative	Total
Positive	77	12	89
Suspicious	15	41	56
Doubtful	16	134	150
Negative	5	1052	1057
**Total**	113	1239	1352
